# Bacterial transmembrane signalling systems and their engineering for biosensing

**DOI:** 10.1098/rsob.180023

**Published:** 2018-04-25

**Authors:** Kirsten Jung, Florian Fabiani, Elisabeth Hoyer, Jürgen Lassak

**Affiliations:** Munich Center for Integrated Protein Science (CiPSM) at the Department of Biology I, Microbiology, Ludwig-Maximilians-Universität München, Martinsried, Germany

**Keywords:** signal transduction, two-component system, ToxR, CadC, KdpD, YehU

## Abstract

Every living cell possesses numerous transmembrane signalling systems that receive chemical and physical stimuli from the environment and transduce this information into an intracellular signal that triggers some form of cellular response. As unicellular organisms, bacteria require these systems for survival in rapidly changing environments. The receptors themselves act as ‘sensory organs’, while subsequent signalling circuits can be regarded as forming a ‘neural network’ that is involved in decision making, adaptation and ultimately in ensuring survival. Bacteria serve as useful biosensors in industry and clinical diagnostics, in addition to producing drugs for therapeutic purposes. Therefore, there is a great demand for engineered bacterial strains that contain transmembrane signalling systems with high molecular specificity, sensitivity and dose dependency. In this review, we address the complexity of transmembrane signalling systems and discuss principles to rewire receptors and their signalling outputs.

## 1. Introduction

Bacteria constantly interact with their surroundings. They identify and actively acquire nutrient resources, sense and respond to environmental stresses and exchange information with other cells, while commensals and pathogens adapt their lifestyles for survival in their hosts. The cytoplasmic (inner) membrane of bacterial cells separates the cytoplasm from the outer world. Therefore, all information from the outside must be transferred across this interface, which contains various sensors that carry out this function.

Based on these natural properties, bacteria can be (re-)programmed to function as biosensors with various applications. Bacterial biosensors could be used to monitor the concentration of toxins or certain process parameters or products and by-products during the production and storage of foods (see reviews [1,2]). Bacterial biosensors have the potential to revolutionize diagnostics and therapeutics, a promising field in synthetic biology. Engineered bacteria have already been used to detect and combat a *Pseudomonas aeruginosa* infection. Such biosensors not only identify the causative agent of pneumonia but they also produce a toxin to kill *P. aeruginosa* after exposure to the pathogen [3]. In the future, bacteria might not only produce drugs, hormones or tumour-killing agents [4] but would also be equipped with sensory systems, so that they move themselves directly to the target site in the body. Bacterial biosensors are self-replicating and less costly devices. They are small and portable, so that they can be used in regions of the world that are far away from modern analytical laboratories.

Despite the promising applications of bacterial biosensors, there are still several limitations. For example, most organic compounds cannot cross the cytoplasmic membrane and therefore biosensors need specific transmembrane signalling systems. Still, the number of thoroughly characterized receptors able to detect external stimuli and transduce the information into a cellular signal is limited. Furthermore, in many bacteria the stimulus–response is not linear, and the degree of output varies from cell to cell within a population. Last but not least, the output needs to be rewired to an easy detectable readout.

**Figure 1.**
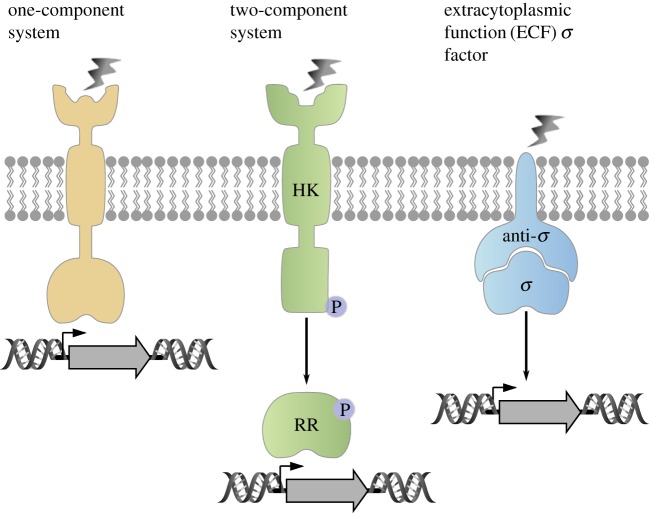
Schematic presentation of the major types of transmembrane signalling systems in bacteria. One-component signalling systems, consisting of sensor and DNA-binding domain (yellow), two-component systems with a membrane-integrated histidine kinase (HK) and a response regulator (RR) (green), and extracytoplasmic function (ECF) sigma factors (*σ*) that will be released from the anti-sigma factor (anti-*σ*) upon stimulus perception (blue).

Natural transmembrane signalling systems are complex. Basically, bacteria use three major types: membrane-integrated one-component systems (ToxR-like receptors), two-component systems consisting of a receptor histidine kinase and a response regulator, and the so-called extracytoplasmic function (ECF) sigma factors [5] (figure 1). In some cases, membrane-integrated transport proteins have secondarily acquired sensory functions [6,7].

Members of the ToxR family all belong to one-component signalling systems. These receptors are bitopic membrane proteins, consisting of a periplasmic sensor domain and an intracellular winged helix-turn-helix DNA-binding domain (figure 1). They do not contain a phosphoryl acceptor domain, and therefore represent the simplest form of bacterial transmembrane signalling systems. The family is named after the main regulator of virulence in *Vibrio cholerae*, ToxR [8].

In two-component systems, the membrane-integrated histidine kinase generally acts as a sensor for various stimuli, and is also responsible for information transfer across the membrane. This process usually results in the autophosphorylation of the protein and the phosphoryl group is subsequently transferred to the cognate soluble response regulator (figure 1), which usually acts as a transcription factor [9]. The number of histidine kinase/response regulator systems varies widely between bacterial species, ranging from 30 in *Escherichia coli* and 36 in *Bacillus subtilis* to 132 in *Myxococcus xanthus* [10]. In chemotactic systems, a soluble histidine kinase perceives the signal(s) conveyed by membrane-integrated chemoreceptors and transduces this information via phosphorylation/protein–protein interaction to the flagellar motor [11].

The ECF sigma factors are small regulatory proteins that bind to RNA polymerase and stimulate transcription of specific genes. Many bacteria, particularly those with more complex genomes, contain multiple ECF sigma factors, and these regulators often outnumber all other types of sigma factors. Little is known about the roles or the regulatory mechanisms employed by the majority of ECF sigma factors. Most of them are co-expressed with one or more negative regulators. Often, these regulators include a transmembrane protein that functions as an anti-sigma factor, which binds and inhibits the cognate sigma factor (figure 1) [12].

In this review, we are describing three examples of the complexity of natural transmembrane signalling systems. Furthermore, we summarize new developments in the rewiring of receptors and the output response [13–15]*.* The intracellular network complexity or synthetic biological gates are not the subject of this article, and interested readers are referred to another review [16].

## 2. CadC: the complexity of a one-component system

One-component systems are widely distributed among bacteria and evolutionarily more ancient than two-component systems. Most one-component systems are soluble cytoplasmic proteins and only 3% are membrane-integrated [17]. Members of this subclass comprise the so-called ToxR family, and share a modular topology consisting of a cytoplasmic N-terminal DNA-binding domain that regulates transcription, a transmembrane helix and a C-terminal periplasmic sensory domain [8]. In addition to ToxR, TcpP and TfoS in *Vibrio cholerae* [18,19], PsaE from *Yersinia pseudotuberculosis* [20], ArnR from *Sulfolobus acidocaldarius* [21] and the pH sensor CadC found in *E. coli*, *V. cholerae* and *V. vulnificus* [22,23] belong to this family.

**Figure 2.**
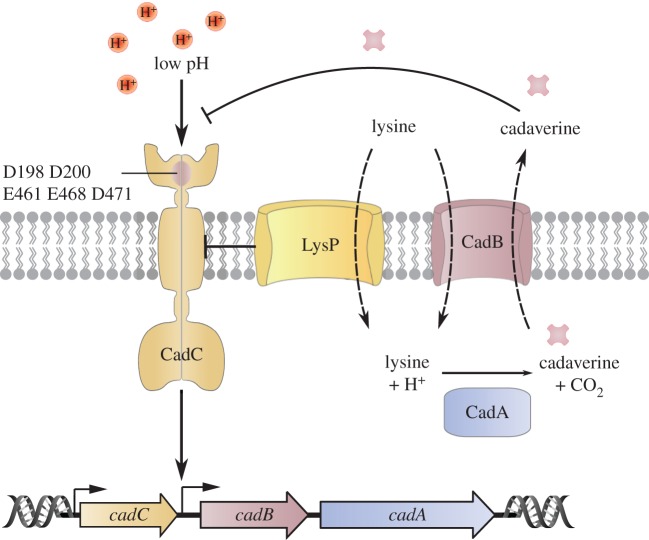
The complex regulation of CadC, a one-component system representative. CadC is the regulator of the *cadBA* operon encoding the lysine decarboxylase CadA and the lysine/cadaverine antiporter CadB. Under non-inducing conditions, the lysine-specific transporter LysP inhibits CadC. When cells are exposed to low pH in the presence of lysine, the interaction between LysP and CadC is weakened, rendering CadC susceptible for protonation and transcriptional activation. The end-product of decarboxylation, cadaverine, binds to CadC and thereby inactivates this receptor.

We have extensively studied the molecular mechanism of the CadABC module in *E. coli,* one of the four inducible, amino acid-specific decarboxylase systems in that species [24]. CadC acts as a homodimeric one-component regulator. CadA is a cytoplasmic decarboxylase, which converts lysine to cadaverine, while CadB is a membrane-integrated lysine/cadaverine antiporter (figure 2) [25–29]. Together, their activities lead to an increase in both internal and external pH, which favours survival of *E. coli* under moderate acid stress and helps to maintain pH homoeostasis [26,30].

CadC is activated by two stimuli, low pH (less than 6.8) and the presence of external lysine (greater than 0.5 mM) [31], which are perceived by different mechanisms [30]. The periplasmic domain of CadC directly senses a decrease in pH. Its crystal structure was solved at a resolution of 1.8 Å and revealed two distinct subdomains: the N-terminal subdomain comprises a mixture of β-sheets and α-helices, and the C-terminal subdomain consists of a bundle of 11 α-helices [32]. A patch of acidic amino acids (D198, D200, E461, E468, D471) is crucial for the detection of alterations in the external pH [30,33]. Presumably, a drop in the external pH leads to protonation of these residues. This, in turn, reduces repulsive forces between the subdomains/monomers and promotes dimer formation of the periplasmic domain, triggering receptor activation [32–34].

The signal perceived at the sensory domain must be transduced via the transmembrane helix to the DNA-binding domain. A linker region comprising 50 amino acids connects the transmembrane helix of *E. coli* CadC with the DNA-binding domain. NMR and bioinformatic analyses revealed that this disordered segment undergoes structural changes that enable the winged helix-turn-helix DNA-binding domain [35] to interact with the target promoter of *cadBA*, leading to expression of the operon [36,37]. Transcriptional activation does not require proteolytic cleavage of CadC, as had previously been suggested [38]. Instead, the full-length, membrane-integrated receptor is capable of binding to P*_cadBA_*, which is an uncommon mode of signal transduction [31,39].

Analogous to pH, lysine was first thought to be sensed directly by CadC [30]. However, it turned out that CadC senses external lysine only in interaction with the lysine-specific permease LysP [30,40,41]. The secondary transporter LysP, composed of 12 transmembrane helices [42], interacts with CadC and transduces the signal to its interaction partner via lysine-dependent conformational changes. Cross-linking studies and bacterial two-hybrid analyses provided proof for direct protein–protein interaction [40]. Further mutagenesis studies identified distinct amino acids in the transmembrane and periplasmic domains of CadC and LysP that are crucial for the hetero-oligomeric interaction and signal transduction [40]. These findings suggest that the interaction of LysP with CadC in the absence of lysine precludes transcriptional activation, whereas the interaction of both proteins is weakened in the presence of lysine and at low pH, leading to conformational changes and destabilization of the hetero-oligomeric interaction.

Furthermore, it has been shown that the products of lysine decarboxylation, CO_2_ [43] and cadaverine, act as feedback inhibitors on CadC [30,31,44]. Cadaverine binds to the periplasmic domain of CadC, thereby switching off *cadBA* transcription [30,44]. Structural analysis of the periplasmic domain of *E. coli* CadC has revealed two binding sites for cadaverine: one lies in a cavity within the periplasmic domain, the other is located at the interface between the two monomers [44].

Lastly, the Cad system of *E. coli* is dependent on translational regulation to limit the number of CadC molecules to 3–5 per cell, which turns out to be essential for an appropriate stress response [45]. Specifically, two motifs, each made up of consecutive prolines, within the unstructured linker cause ribosome stalling, which can only be alleviated by the bacterial translation elongation factor P (EF-P). In fact, expression of a *cadC* variant in which the proline codons that induce stalling were mutated (making synthesis of the variant (CadC-PPPIP/AAAIS) independent of EF-P) led to an increase in the steady-state level of the protein to 11–14 copies per cell. This increase, in turn, alters the balance between CadC and LysP and ultimately results in aberrant activation of P*_cadBA_* [45].

## 3. KdpD/KdpE: dual sensing in a canonical two-component system

Histidine kinase/response regulator systems significantly outnumber other known bacterial transmembrane signalling systems in nature. Although the basic mechanism of action of histidine kinase/response regulator is quite well understood [46], the primary stimulus sensed by the histidine kinase is often less well defined. Generally, stimuli detected by histidine kinases can be grouped into chemical parameters, such as organic compounds (e.g. C4 dicarboxylates, citrate, autoinducers), inorganic compounds (Mg^2+^, H^+^, K^+^) and gaseous ligands (e.g. O_2_, N_2_), and physical parameters, such as osmolarity/turgor, light and temperature [10]. Stimuli are perceived via periplasmic/extracellular sensing domains that are characterized by specific folds. The most common domains belong to the PAS (Per-ARNT-Sim), CHASE (cyclase/histidine kinase-associated sensing extracellular), four-helix bundle (4HB) and NIT (nitrate and nitrite-sensing) classes [47].

**Figure 3.**
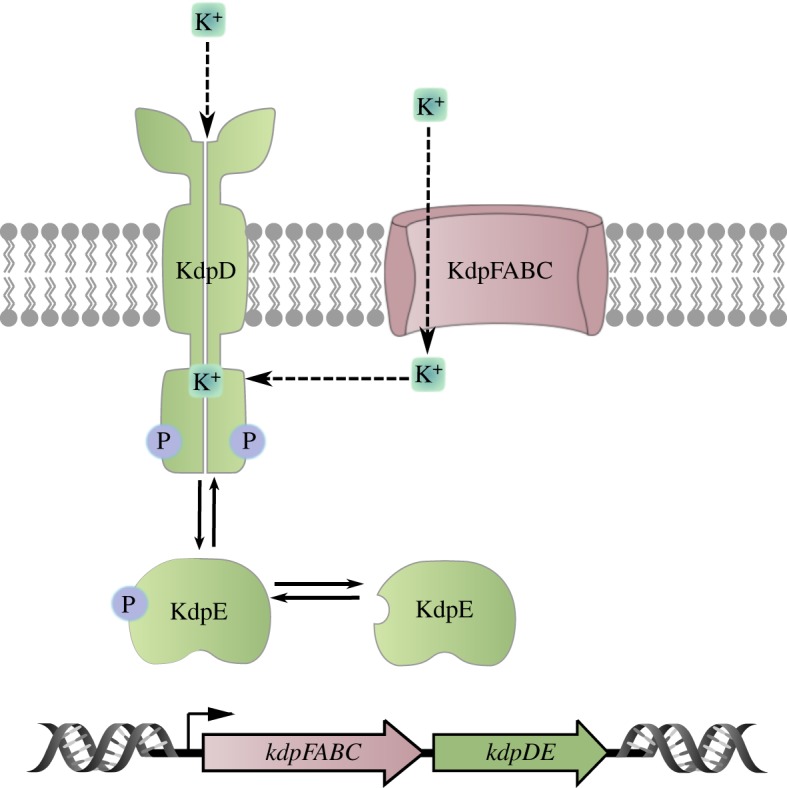
Schematic of the Kdp regulation system. The bifunctional receptor histidine kinase KdpD acts as both an autokinase (including phosphotransferase) and phosphatase for the response regulator KdpE. Phosphorylated KdpE activates expression of the genes encoding the high-affinity K^+^ transporter KdpFABC. KdpD autokinase activity depends on the external K^+^ concentration, and the phosphatase activity is influenced by the internal K^+^ concentration.

It is often difficult to identify the primary stimulus for a receptor, as exemplified by the histidine kinase KdpD which, together with the response regulator KdpE, controls the expression of a high-affinity K^+^-uptake system in many bacteria. K^+^ is the most abundant cation in all living cells, and especially in bacteria it is crucial for the regulation of cell turgor and intracellular pH and for the activation of several enzymes [48–50]. To ensure a sufficient supply of K^+^, most bacteria have more than one K^+^-uptake system. For example, *E. coli* has at least three such systems, the constitutively expressed systems Trk and Kup, and the inducible high-affinity K^+^-uptake system KdpFABC [51]. The genes *kdpF, kdpA, kdpB* and *kdpC* form an operon that codes for four inner membrane proteins. The *kdp* operon is induced when *E. coli* is grown under K^+^ limitation, or lacks the major K^+^ transporter Trk or has an increased need for K^+^ when under hyperosmotic stress [51]. Under all these conditions, the membrane-integrated histidine kinase KdpD autophosphorylates and transfers the phosphoryl group to the cytoplasmic transcriptional (response) regulator KdpE, resulting in the induction of the *kdp* operon (figure 3). KdpD also exhibits phosphatase activity towards phosphorylated KdpE, which switches the signalling cascade off [52]. In K^+^ limited conditions, KdpD/KdpE activation correlates inversely with the external K^+^ concentration up to 0.5 mM, at which point an additional amplification of the expression level is observed [53].

The pattern of induction suggests that cells detect the need for K^+^ rather than measuring the absolute K^+^ concentration. Because K^+^ plays a major role in maintaining turgor (the difference in osmotic pressure across the inner membrane), it was first proposed that expression of the *kdp* operon is dependent on turgor, and that KdpD functions as a sensor of turgor [52,54]. However, subsequent measurements of cytoplasmic volume ruled out changes in turgor as the primary stimulus for KdpD [55]. Alternatively, internal K^+^ levels and/or K^+^ uptake rates, or alterations in lipid composition have been discussed as putative primary stimuli for KdpD [56]. Finally, phosphorylation of KdpE by KdpD is inhibited *in vitro* by increasing K^+^ concentrations [57,58]. As K^+^-binding sites in the protein could not be predicted, it was even debated whether KdpD responds to alterations in the extra- or intracellular K^+^ concentration [57,59].

Recently, we solved this puzzle and identified KdpD as a dual K^+^-sensing histidine kinase [60]. We found that both enzymatic activities of KdpD are directly influenced by K^+^ (figure 3). When the extracellular K^+^ concentration is high (greater than 5 mM), the ion binds to an externally accessible site, and this leads to the inhibition of the autokinase activity. At the same time, intracellular K^+^ is sensed by the C-terminal cytoplasmic domain and stimulates the phosphatase activity. Consequently, KdpD acts as a phosphatase on phosphorylated KdpE, and production of the high-affinity K^+^ transporter is prevented. When environmental levels of K^+^ fall below the threshold for autokinase activation, *kdpFABC* expression is initiated; however, as long as the intracellular K^+^ concentration remains high, the KdpD phosphatase activity remains stimulated. Under these conditions, the intracellular response is attenuated for as long as the high intracellular K^+^ concentration is sufficient for the operation of all cellular processes. The longer the cells are exposed to K^+^ limitation or extreme K^+^ limitation, the greater is the drop in intracellular K^+^. Eventually, the phosphatase activity is no longer stimulated and larger fractions of KdpE become phosphorylated, resulting in maximal production of KdpFABC.

This dual-regulation mechanism allows *E. coli* and other bacteria not only to respond to impending limitation by sensing the extracellular K^+^ concentration but also to regulate the activation level in response to changing intracellular demand for K^+^. These experimental studies were complemented by mathematical modelling [60]. Using simulations, the dual-sensing strategy was compared to strategies involving sensing of only one K^+^ pool under variation of both environmental K^+^ and growth rate. The dual-sensing strategy clearly outcompeted single-sensing strategies, because it ensures cellular K^+^ homeostasis under widely differing conditions.

Dual sensing thus emerges as a highly optimized regulation strategy. The key advantage of this strategy is that it confers on cells the ability to directly sense changes in both the supply of and demand for the limiting resource. It is, in fact, analogous to strategies that are widely used in control engineering, e.g. modern heating systems work with both exterior and interior thermometers to ensure constant room temperature [61].

Owing to the enormous advantage of dual receptors for the maintenance of cellular homeostasis, this mechanism might have evolved much more commonly. For engineering purposes, the use of dual receptors deserves to be taken into consideration, but in cases where a linear stimulus–response behaviour is required, the internal sensing part should be removed or replaced.

## 4. BtsS/BtsR: the many regulatory layers of a two-component system

One of the most crucial factors for growth is the ability to sense the presence and type of nutrients available in the environment in order to adapt metabolism for optimal exploitation. The BtsS/BtsR two-component system (previously known as YehU/YehT) has recently been identified as a high-affinity sensory system that is able to detect extracellular pyruvate concentrations as low as 50 µM [62].

**Figure 4.**
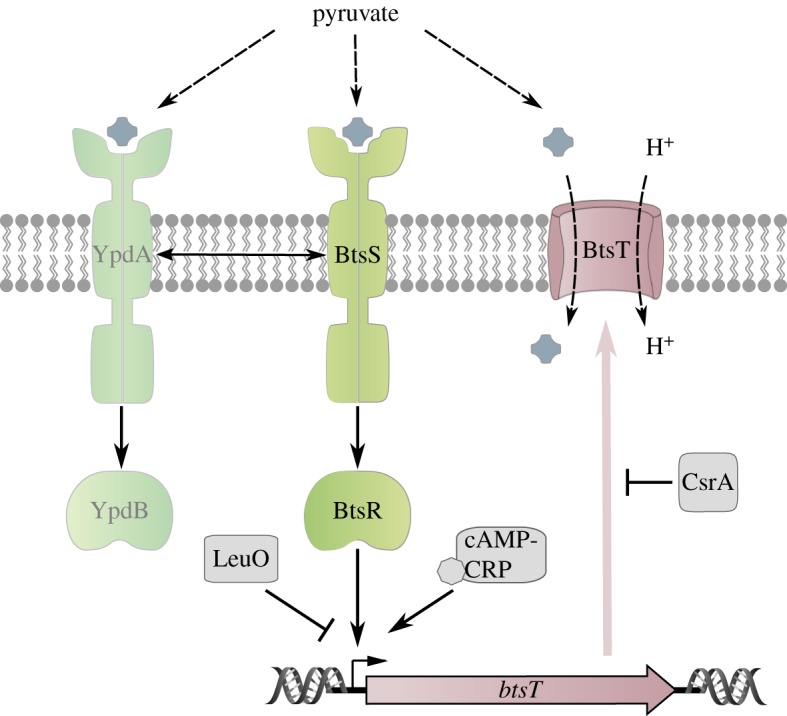
Schematic of the pyruvate-sensing BtsS/BtsR/YpdA/YpdB network. The scheme summarizes the regulatory network associated with signal transduction by the BtsS/BtsR system, the influence of the YpdA/YpdB system and the global regulators cAMP-CRP, LeuO and CsrA.

BtsS/BtsR is highly conserved among bacteria and orthologues can even be found in several plants, animals and human pathogens [62]. This two-component system is composed of the sensor histidine kinase BtsS, a member of the LytS histidine kinase family, and BtsR, a representative of the LytTR family of response regulators (figure 4) [63]. The input domain BtsS is composed of the Lyt domain with six transmembrane helices, and a cytoplasmic GAF domain. GAF domains fold similarly to PAS domains and are capable of binding small ligands such as cGMP, formate, 2-oxoglutarate and aromatic compounds, but they are also involved in protein–protein interactions [64]. The response regulator BtsR is composed of a CheY-like receiver domain, with the conserved aspartate D54, and a LytTR-type DNA-binding domain with a 10-stranded β-fold [65].

BtsS/BtsR regulates a single target gene, *btsT* (previously known as *yjiY*) [66]. BtsT has 18 predicted transmembrane domains and is a member of the CstA transporter family. Expression of *E. coli btsT* is induced under nutrient limitation and in the presence of pyruvate [62]. Not surprisingly then, BtsT was shown to import pyruvate in symport with H^+^ [67].

Although the role of BtsS as a pyruvate sensor has been clearly demonstrated, phenotypical analysis in both *E. coli* and *Salmonella enterica* failed to detect any significant difference between the *btsSR* deletion mutant and the parental strain. Neither growth, stress adaptation, antibiotic resistance, biofilm formation, invasive capacity or phage susceptibility was affected [63,68]. Only increases in resistance to crystal violet and oxaloacetate were observed upon overexpression of BtsR [69].

Notably, BtsS/BtsR forms a functional network with the paralogous YpdA/YpdB two-component system (figure 4) and its sole target, a transporter of so far unknown function called YhjX [70]. Like BtsS/BtsR, the YpdA/YpdB system responds to pyruvate, albeit with lower affinity [66].

Recently, a single-cell analysis showed that in the absence of both systems, the frequency of stochastically formed persisters significantly increases. Moreover, only half of the cells were able to cope with the metabolic burden when challenged to overproduce various proteins. This suggests that BtsS/BtsR together with YpdA/YpdB balances the physiological state of all cells within the population by uptake of nutrients [71].

The identification of pyruvate as both a ligand for BtsS and a substrate for BtsT points towards a simple and straightforward stimulus–response mechanism. However, *btsT* expression is modulated by an intricate regulatory network which could complicate signal cascade engineering (figure 4). First, *bstT* expression is controlled by the cAMP–CRP complex, and is subject to catabolite repression [63]. Second, it is repressed by the LysR-type transcriptional regulator LeuO [72]. Third, BstT synthesis is post-transcriptionally controlled by the carbon storage regulator protein CsrA [73]. Fourth, BtsT levels are affected by the YpdA/YpdB system, as *btsT* expression significantly decreases in the absence of either YpdA, YpdB or YhjX [70]. Nonetheless, the latter proteins are found only in a subset of the species that encode the BtsS/BtsR pair. Notably, they are absent in some close relatives of *E. coli*, such as *S. enterica*. Fifth, *btsT* expression exhibits a high degree of cell-to-cell variability. Analysis at the single-cell level showed that *bstT* transcription was highly heterogenous even at saturating pyruvate concentrations (20 mM) [71]. Finally, there are indications that BtsS/BtsR and the homologous LytS/LytT system in *Bacillus subtilis* are also influenced by internal stimuli, e.g. the concentration of pyruvate or malate [62,74].

The centrality of carbon metabolism and pyruvate's pivotal role highlight the importance of the BtsS/BtsR system. Indeed, the phosphoenolpyruvate–pyruvate–oxaloacetate node is the point at which metabolism switches between gluconeogenesis, oxidation and fermentation. Pyruvate is, therefore, crucial for bacterial fitness and hence a target of particular interest for metabolic engineering [75]. This growing interest recently led to the characterization of a novel LytS/LytT-inducible, pyruvate-specific transporter in *B. subtilis* [74]. The authors considered their findings to be particularly useful in the quest for ways to rewire metabolic pathways in order to efficiently produce enzymes or other chemicals [76]. Furthermore, pyruvate is essential for the virulence of many intracellular pathogens, including *L. monocytogenes* and *S. enterica* [77,78]. Similarly, in *S. aureus* and *Yersinia pseudotuberculosis*, pyruvate metabolism controls host colonization and virulence [79,80]. Finally, in most human tumour cells, glycolysis and fermentation are highly upregulated, and oxidative phosphorylation is downregulated. This phenomenon, known as the Warburg effect, is responsible for the increased concentrations of pyruvate in the cytosol of cancerous cells [81]. Understanding pyruvate sensing in bacteria might, therefore, help to improve the specificity or efficiency of promising treatment strategies, such as bacterially based tumour-targeting cancer therapy.

Although a more detailed understanding of its sensory mechanisms, complex regulation and cell-to-cell variability is required, the characterization of BtsS as the first high-affinity pyruvate sensor could lead to major improvements in metabolism engineering, pathogenesis control and tumour monitoring or therapy.

## 5. Rewiring receptors and signalling outputs

In the previous sections, we have discussed three natural one- and two-component receptors and their signalling circuits. We will now describe tools and principles that can be used to rewire transmembrane signalling systems.

**Figure 5.**
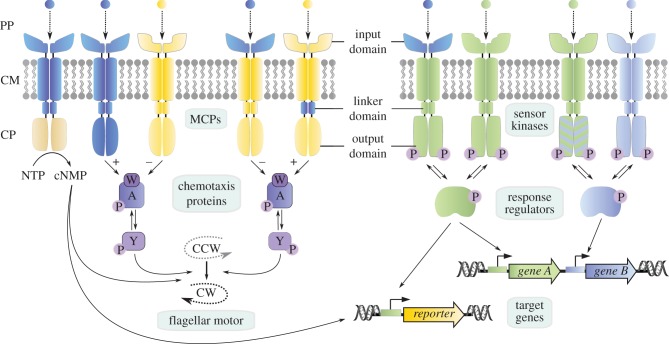
Principles to rewire transmembrane signalling systems. Membrane-integrated methyl-accepting chemotaxis proteins (MCPs, left part) are generally composed of two modules: an input domain in the periplasm (PP) and in the cytoplasmic membrane (CM), which is responsible for ligand binding and signal transduction, and an output domain in the cytoplasm (CP), which induces a cellular response. Both domains are connected by a linker domain. Whereas input domains are highly diverse, variation in the output domains is rather limited. There are three common schemes for an output: nucleotide cyclase activity (NTP = nucleotide triphosphate → cNMP = cyclic nucleotide monophosphate) (outermost left); alterations of the direction of the flagellar motor (CCW = counter clockwise, CW = clockwise rotation) including the formation of a ternary complex between MCPs/CheW(W)/CheA(A) and (de-)phosphorylation of CheY(Y) (innermost left), and transcriptional regulation (right). Sensor kinases perceive a stimulus and transduce the signal via phosphorylation to a response regulator that acts as transcription factor of natural or reporter genes. The modular design of transmembrane signalling systems allows the generation of chimeric receptors in which the input, the linker or the output domain is replaced (domain colour switch). Sensor kinases can be rewired by amino acid replacement (blue/green stripes) to allow activation of a non-cognate response regulator.

The modular design of bacterial membrane-bound sensory systems enables researchers to create novel input–output combinations by generating chimeras (figure 5). Such hybrid proteins are of particular interest when they alter the natural target and with it the output, e.g. from transcription to motility as in the case of bacterial chemoreceptors (figure 5). These methyl-accepting chemotaxis proteins (MCPs) generally consist of an input and an output module [82]. The conserved output module is built of a dimeric four-helix bundle composed of two symmetrically arranged coiled coils [83,84]. By contrast, the input domain can be highly diverse, which allows for the integration of a broad spectrum of external signals, acting either as attractants or repellents [85]. In *E. coli*, binding of a ligand to the input domain induces a conformational change in the last periplasmic helix, which is propagated into the second transmembrane helix [82,86–89]. Notably, similar rearrangements have also been reported for histidine kinases [90], indicating a common mechanism of transmembrane signalling despite the structural diversity of ligand-binding input domains (figure 5). Indeed, several research groups have successfully constructed functional chimeric receptors. For example, a team led by Mike Manson engineered a repellent response to nitrate and nitrite by fusing the ligand-binding, transmembrane and linker domains of the histidine kinase NarX to the *E. coli* aspartate-responsive MCP Tar [91]. Bi *et al*. [92] have described several other active Tar hybrids in which, for instance, the four-helix-bundle domain [93] of the nitrate/nitrite-sensing histidine kinase NarQ of *E. coli* [94] was fused to Tar. A second Tar hybrid was generated with the helical bimodular (HBM) domain [95] of the *P. putida* MCP McpS, which recognizes TCA cycle intermediates and acetate [96,97], while a third uses the nitrate-responsive NIT domain [98] of the putative chemoreceptor ECA0434 from *Pectobacterium atrosepticum*. This group also fused Tar to single and double PhoQ-DcuS-CitA (PDC) domains [99–102], and has generated several other examples of Tar hybrids with double PDC domains [103,104]. In conclusion, these examples validate a general strategy for constructing functional hybrid chemotaxis receptors by combining the cytoplasmic MCP output domain with sensory domains for diverse inputs (figure 5). This not only is useful for applications in synthetic biology but also enables ligand identification and binding studies [92].

Conversely, chemoreceptor input domains can be fused to distinct output domains, as illustrated by Taz, a hybrid in which the Tar sensing domain is coupled to the histidine kinase EnvZ of *E. coli* [105]. Depending on external osmolarity, EnvZ, together with its cognate response regulator OmpR, regulates the transcription of the porin-coding genes *ompF* and *ompC* [106,107]. Like Tar, EnvZ contains two transmembrane helices and has the same topological orientation. The original construction of functional Taz hybrids made use of an *Nde*I restriction site present downstream of the sequence coding for the second transmembrane helix in both Tar and EnvZ [108]. Therefore, it was possible to engineer a signalling system that uses aspartate as a precisely adjustable stimulus to trigger a transcriptional response. This subsequently allowed a detailed analysis of two-component signal transduction [105].

Along the same line, the above described one-component-system CadC was used to generate a functional hybrid responding to caffeine as external stimulus [14]. CadC activation requires dimerization via its periplasmic pH sensing domain [32,34,36]. Accordingly, a protein variant lacking this input domain can no longer induce *cadBA* transcription. By fusing the CadC DNA-binding domain to a single-domain camelid antibody V_H_H [109], Chang *et al*. [14] achieved ligand-induced dimerization and could, in turn, activate the P*_cadBA_* promoter. This exemplifies how modular receptor design based on split-DNA-binding domains combined with the versatility of antibody-based ligand detection can greatly expand the sensory repertoire in bacteria.

When generating functional chimeric proteins such as Taz, careful selection of the point of fusion is especially crucial, as signal transduction has to remain intact. Accordingly, the structural elements that transmit the sensory information from the input domain to the output domain are of great importance. Probably the best-studied and most frequently occurring linker in bacterial signalling systems is the so-called HAMP domain [110]. The HAMP domain is named after its occurrence in histidine kinases, adenylyl cyclases, methyl-accepting chemotaxis proteins, and some phosphatases [111,112]. Structurally, two 16-residue amphiphilic helices are connected by a segment comprising 14 or 15 amino acids. HAMP domains can apparently adopt either of two conformations in which the four-helix bundle is either loosely [113] or tightly packed [114]. Changes in packing might modulate the signalling output, thus determining the output status of the protein [110]. The increasing number of reports on functional chimeras [92,103,115] indicates that the HAMP linker provides a reliable and conserved transmembrane signalling mechanism for synthetic sensory receptors.

Another connector frequently found between input and output domains is the ‘signalling helix’ or (S)-helix, which can be up to 50 amino acids long [116]. This type of helix seems to form a novel parallel coiled-coil element that differs markedly from the other helical elements in signalling proteins. The S-helix can connect diverse N-terminal input domains to various C-terminal domains, such as cNMP cyclases, histidine kinases, PP2C phosphatases, NtrC-like AAA^+^ ATPases and diguanylate cyclases. A unique conserved constellation of polar residues is located at the dimer interface within the central heptad of the coiled coil, which is thought to act as a switching element. Like HAMP, the S-helix can be used to generate functional chimeras, as was demonstrated by Winkler *et al.* [117]. The authors of that study fused the adenylyl cyclase CyaG from *Arthrospira maxima* to the Tsr chemoreceptor and obtained a serine-responsive hybrid. Specific deletions in the S-helix switched the effect of the signal from attractant to repellent, thus demonstrating that targeted modification of connector modules can be used to alter the polarity of the cellular response. Notably, the pH sensor CadC described above uses neither a HAMP domain nor an S-helix to transduce the detection of an acidic environment into a transcriptional readout. Instead, the signal is transduced via an unstructured linker, approximately 50 amino acids long [36]. Although such linkers also form part of other receptors [21], the mechanism by which they transduce signals remains poorly understood. Nevertheless, deletion of the unstructured linker in CadC resulted in a receptor variant which produced a reversed signalling output [36]. These results underline the pivotal importance of connector elements for the engineering of chimeric transmembrane receptors.

Apart from the construction of chimeric proteins, two-component signal transduction systems provide another way to engineer a desired response (figure 5). The specificity of the two components is determined by a limited set of amino acids in each protein, which was initially identified by a computational approach based on the analysis of co-variation in large sets of cognate pairs [118,119]. The replacement of these amino acids in EnvZ rewired its specificity and allowed robust phosphorylation of response regulators other than OmpR [118]. Three substitutions were sufficient to change the specificity [120]. Notably, however, the mutational effects are not simply additive but are highly context dependent.

However, engineering of the transmembrane signalling protein alone does not result in a novel biosensory pathway, because the design of the output is essential for monitoring the response (figure 5). As described above, a chemotactic response, as exemplified by the Tar or Tsr chimeras, is a very useful readout. Such a response can be measured using various chemotaxis assays, e.g. tracking of bacterial locomotion in microfluidic devices, or FRET analyses [121]. Alternatively, the signalling input can be coupled to an enzymatic activity, as illustrated by the Tsr adenylate cyclase hybrid [117]. Similarly, in certain bacterial signalling systems, including two-component systems, responses are coupled to cyclic di-GMP synthesis and degradation [122–124]. In bacteria, the intracellular level of cyclic di-GMP regulates the switch between a sessile and motile lifestyle, for instance [125,126]. Accordingly, biofilm formation can be used as a reporter readout for this class of transmembrane signalling systems.

The most frequent response to an external stimulus, however, is an alteration in transcriptional activity (figure 5), which has been extensively studied for LacI and AraC regulators [124,127–129]. Reporter genes coding for the β-galactosidase LacZ [128,130] or the luciferase system LuxCDABE enable conversion of the external stimulus into a visual and quantifiable response. When fluorescent reporters such as GFP or mCherry are used, a readout is even possible at the single-cell level [71]. Alternatively, a transcriptional response can be triggered by interactions with the RNA polymerase that prevent or stimulate its recruitment to a certain promoter. Examples of this approach include the alpha-proteobacterial response regulator PhyR [131,132] or the two-component regulator CbrB in *Pseudomonas* [133,134].

By combining several of the principles outlined above, a recent study reported the engineering of RGB colour vision into *E. coli* [135]. The RGB programme consists of four subsystems. The first is an array of light sensors, which respond to different wavelengths. Specifically, red and green light sensing is based on phytochromes with phycocyanobilin chromophores, whereas the blue light sensor contains a flavin mononucleotide. The second subsystem – the ‘circuit’ – processes the signals to integrate them or execute a dynamic response. Third, the so-called resource allocator acts as a connector between the circuit output and the ‘actuators’, the fourth subsystem. The spectral response of the engineered RGB system was measured by using fluorescent reporters or enzymes that generate coloured pigments. Fernandez-Rodriguez *et al.* [135] were able to produce ‘colour photographs’ of grown bacteria on plates. This is a perfect example of the potential of rewired prokaryotic signalling cascades for synthetic biology.

## 6. Concluding remarks

This complexity in sensing, the multiple inputs during information processing and finally the overall complex cellular network might well be an obstacle to the reconfiguration of transmembrane signalling systems for use in synthetic biology. At all events, it underlines the need to obtain a comprehensive molecular picture of any such sensory system before embarking on its modification for specific applications. Nevertheless, transmembrane signalling systems hold great promise to be used in novel biosensors with industrial and clinical applications including monitoring and therapy.

## Data accessibility

This article has no additional data.
